# Supplementary material for: A robust deep learning workflow to predict CD8 + T-cell epitopes
Source: Genome Med. 2023 Sep 13;15:70. doi: 10.1186/s13073-023-01225-z (PMC10498576; doi:10.1186/s13073-023-01225-z)
Supplement: Supplementary file 1 — Additional file 1: Fig. S1. Screnshot of TRAP web application. Fig. S2. Cross-species variation and HLA-bias. Fig. S3. Effect of anchor and contact positions on peptide immunogenicity. Fig. S4. Intra vs. Inter-HLA variability. Fig. S5. Pathogenic and self-antigen datasets. Fig. S6. Sequence patterns discriminating epitopes versus non-epitopes. Fig. S7. Out-of-distribution detection. Fig. S8. Benchmark TRAP performance. [file 13073_2023_1225_MOESM1_ESM.pdf]

Supplementary Figures to
A robust deep learning workflow to predict CD8+ T-cell epitopes

Fig S1

TRAP: Deep learning platform for CD8+ T-cell epitope prediction

Self-antigen model

Drag and Drop or Select Files

Making predictions...

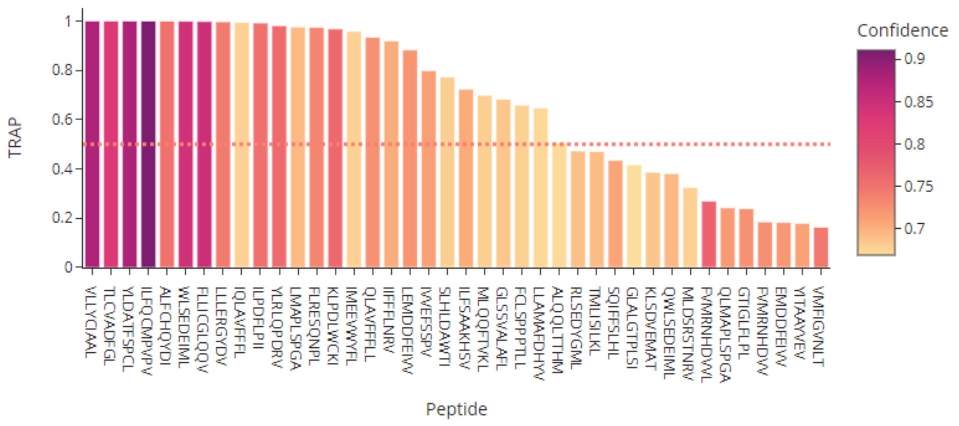

gbm_example_test_data.csv

EXPORT

Peptide	nlog2Rank	TRAP	MCDropout	Confidence
VLLYCIAAL	-0.614	0.99988	6.58	0.871
TLCVADFGL	-0.404	0.99987	5.362	0.834
YLDATFSPCL	-0.019	0.99985	6.662	0.874
ILFQCMPVPV	-0.568	0.99981	7.448	0.897
ALFCHQYDI	0.199	0.99947	2.636	0.753
WLSEDEIML	4.658	0.99886	5.756	0.846
FLLICGLQQV	0.907	0.99786	5.844	0.849

Supplemetnary Figure 1. Screnshot of TRAP web application. TRAP has been developed as a user-friendly web application, where users can enter their peptide list and select the model of interest (pathogenic or self-antigen), and the application will compute the prediction scores along with its confidence. The users can export figure and/or table for their downstream applications. A step-by-step tutorial on how to use the web application as well as the minimal input data requirement is included in the Supplementary document.

Fig S2

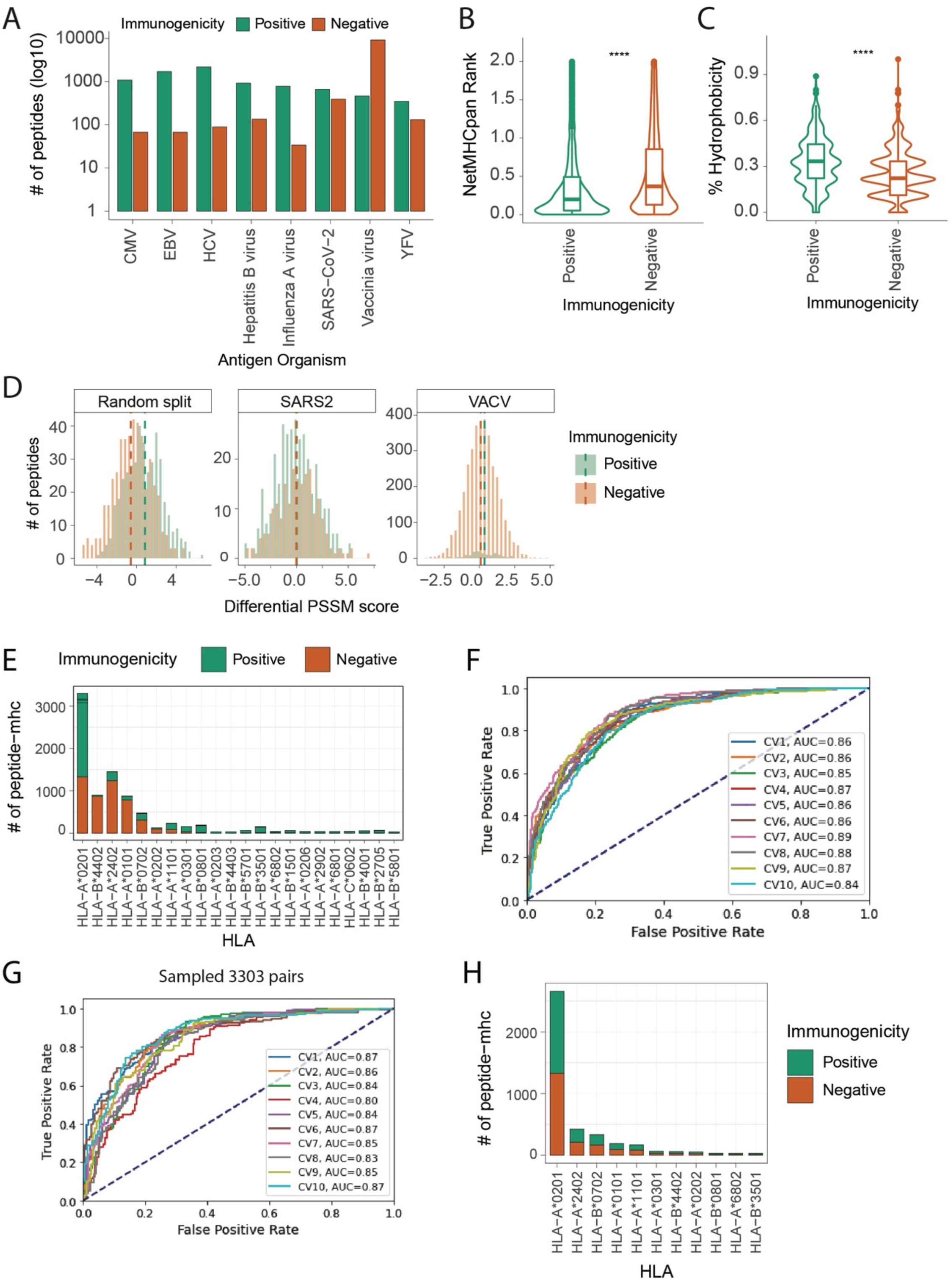

Supplementary Figure 2. Cross-species variation and HLA-bias. A. Statistics of peptides in pathogenic data by their species of origin. B. Distribution of MHC binding rank scores (-log2 transformed) predicted by NetMHCpan 4.0 on pathogenic peptides. C. Distribution of hydrophobicity (i.e. the proportion of hydrophobic amino acids, A, V, L, M and W) of the pathogenic peptides. D. Distribution of differential position specific scoring matrix (dPSSM) score. The dPSSMs were first generated by using training datasets i.e. 90% random data, non-SARS2, non-VACV peptides and were used to predict dPPSM scores on the test dataset i.e. 10%

random data, SARS2 and VACV peptides. E. Statistics of peptides in DeepImmuno training dataset. F. Performance of DeepImmuno. The ROC curve was reproduced as published in the original publication by 10-fold cross-validations. G. ROC curve showing DeepImmuno performance on randomly sampled 3303 peptide-HLA pairs (i.e. equivalent number of peptide-HLA pairs as HLA*02:01) to demonstrate that reduced accuracy of single-HLA predictions is not due to lower number peptides in the datasets. H. Statistics on the number of peptides per HLA after down-sampling to balance the number of epitopes and non-epitopes.

Fig S3

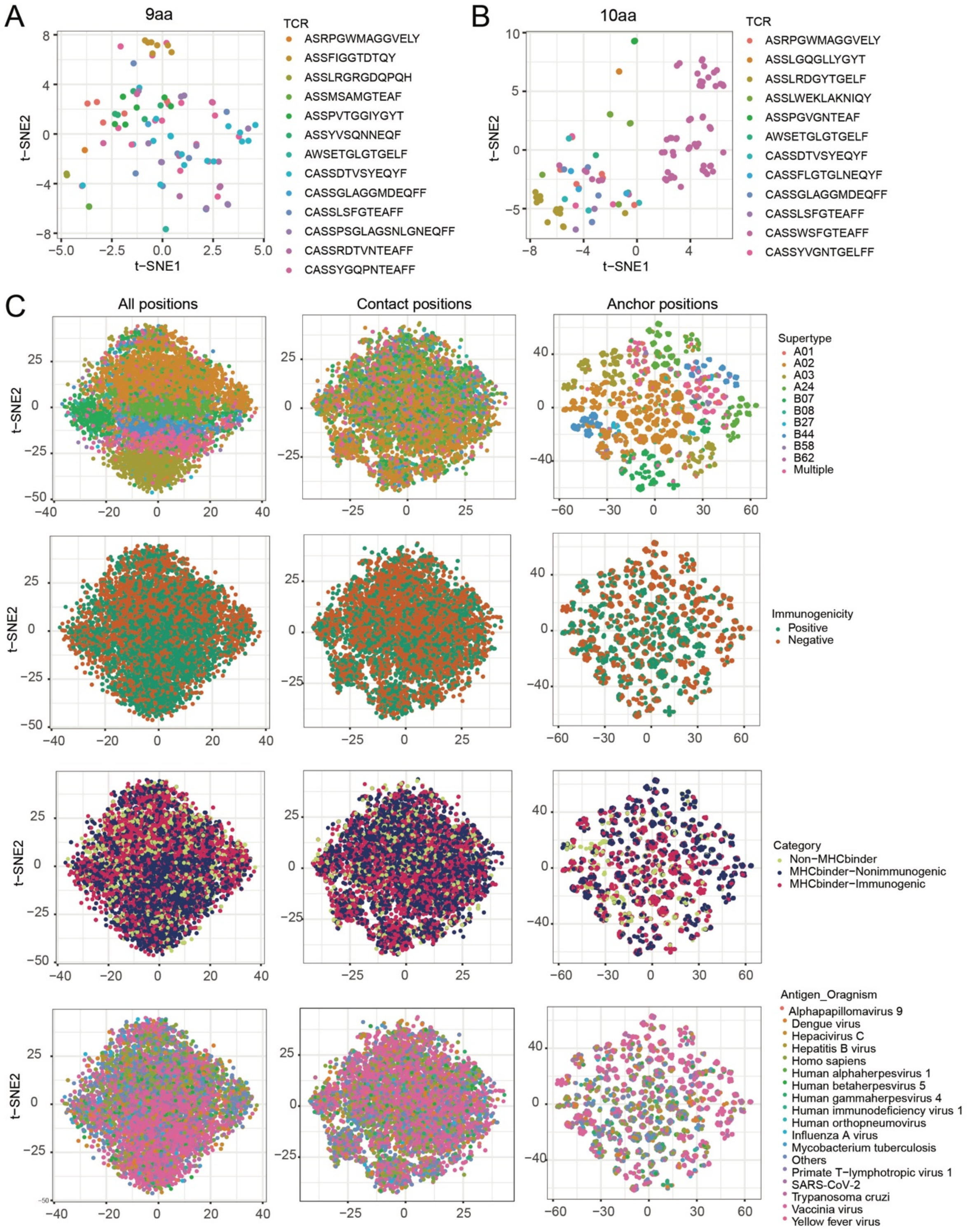

Supplementary Figure 3. Effect of anchor and contact positions on peptide immunogenicity. A-B. t-SNE embedding of peptides represented by peptide-wide descriptors and amino acids at anchor positions, coloured by cognate TCRs for 9aa peptides (A) and 10aa peptides (B). C. t-SNE embeddings of peptides represented by peptide-wide descriptors and position-specific amino acid descriptors, coloured by supertype, immunogenicity, MHC binding and source organism of each peptide. The t-SNEs contain amino acid residues at

different positions, in which left plots contain amino acids at all positions, middle plots contain amino acids at contact positions and right plots contain amino acids at anchor positions.

Fig S4

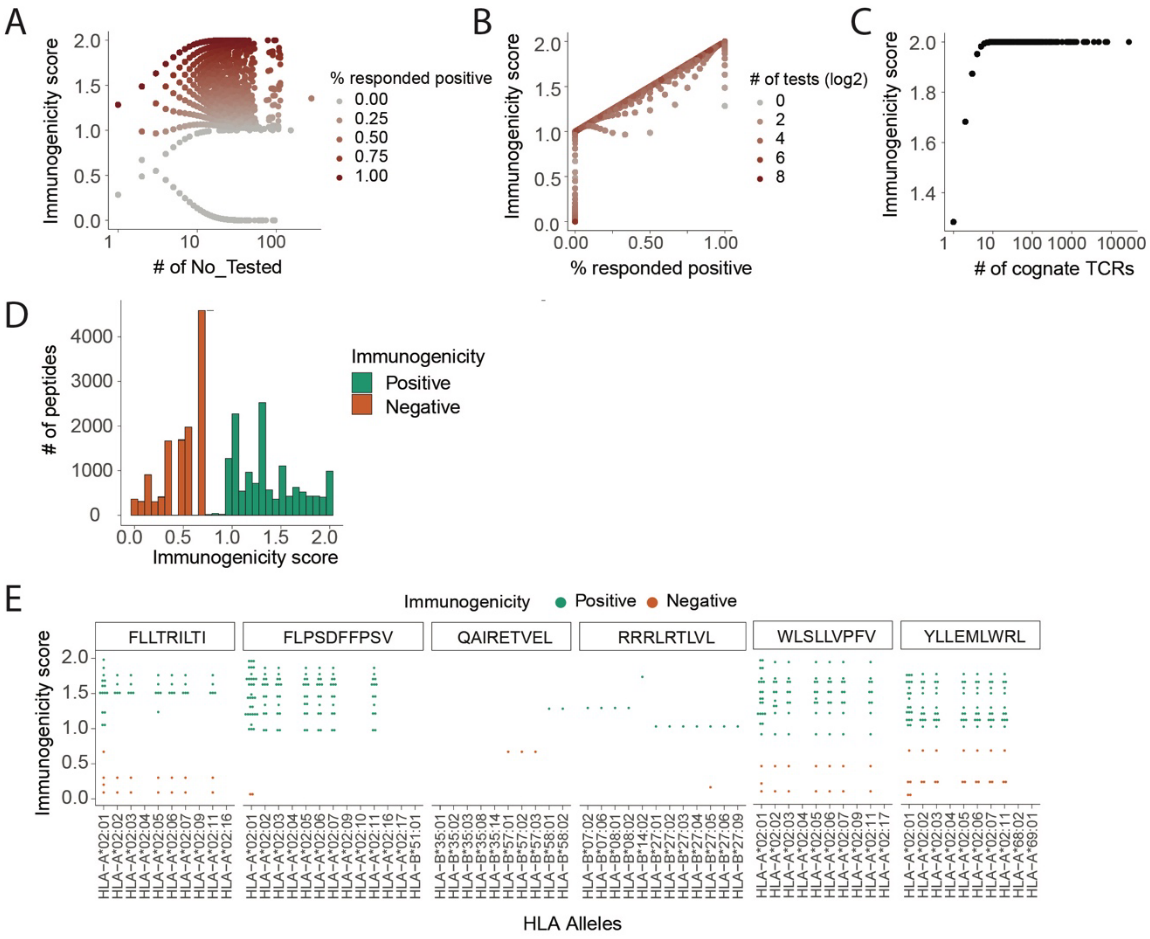

F

Peptide	Immunogenicity	MHC Restriction	Antigen Organism	# Tested	# Responded	Response frequency	In vitro stimulation	Assay	Assay group	Effector cell culture condition	Antigen presenting cells
GILGFVFTL	Positive	HLA-A*02:01	Influenza A virus				Restimulation in vitro	51 chromium	cytotoxicity	Cell Line / Clone	B cell
GILGFVFTL	Positive	HLA-A*02:01	Influenza A virus				Restimulation in vitro	ELISPOT	IFNγ release	Direct Ex Vivo	PBMC
GILGFVFTL	Positive	HLA-A*02:01	Influenza A virus				Restimulation in vitro	multimer/ Tetramer	qualitative binding	Short Term Restimulated	
GILGFVFTL	Positive	HLA-A*02:01	Influenza A virus	6	6	100		ICS	CXCL9/MIG release	Direct Ex Vivo	PBMC
GILGFVFTL	Positive	HLA-A*02:01	Influenza A virus	5	2	40		ELISPOT	IFNγ release	Direct Ex Vivo	PBMC
GILGFVFTL	Positive	HLA-A*02:01	Influenza A virus	1	1	100	Restimulation in vitro	51 chromium	cytotoxicity	Cell Line / Clone	B cell

Supplementary Figure 4. Intra vs. Inter-HLA variability. A-C. Convert binary to quantitative positivity score. The positivity score is computed using the number of tests conducted (A), the percentage that responded positive (B) and the number of cognate TCRs (C). D. Distribution of positivity score. In essence, for positive peptides, the higher the positivity score, more likely the peptide is to be immunogenic. For negative peptides, the lower the score, more likely the peptide to be non-immunogenic. E. Example of peptides having higher intra-HLA variation than inter-HLA variation. F. Sources of intra-HLA variation. The causes of intra-HLA variation include biological and technical factors, such as different assays, effector population and antigen-presenting cells in characterizing the immunogenicity of peptides bound on the same HLA. '-' means information is not available. For peptide-HLAs without supporting information, positivity scores were denoted as binary values.

Fig S5

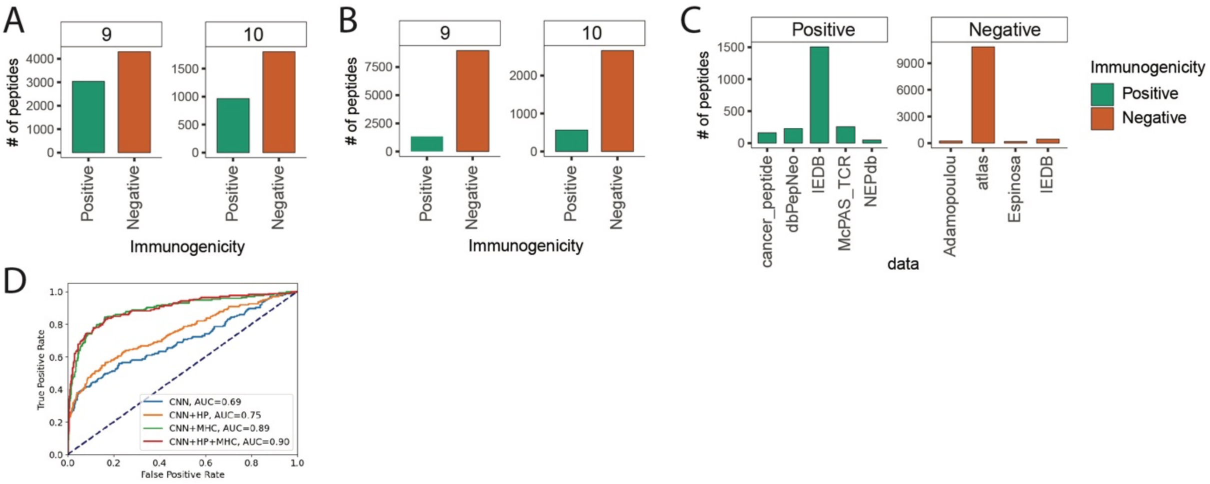

Supplementary Figure 5. Pathogenic and self-antigen datasets. A. Statistics on the number of peptides in the pathogenic dataset. B. Statistics on the number of peptides in the self-antigen dataset. C. Statistics on the number of self-epitopes and HLA-I ligands expressed in thymus, retrieved from publications and databases. D. ROC curve comparing the performance of TRAP when immunogenicity hallmarks – MHC binding rank score and/or hydrophobicity – are added to a peptide sequence-based 1D CNN model for self-peptides. The self-antigen model was trained on self-epitopes and non-epitopes that had been pre-filtered using a relaxed MHC binding relaxed threshold (NetMHCpan rank = 10).

Fig S6

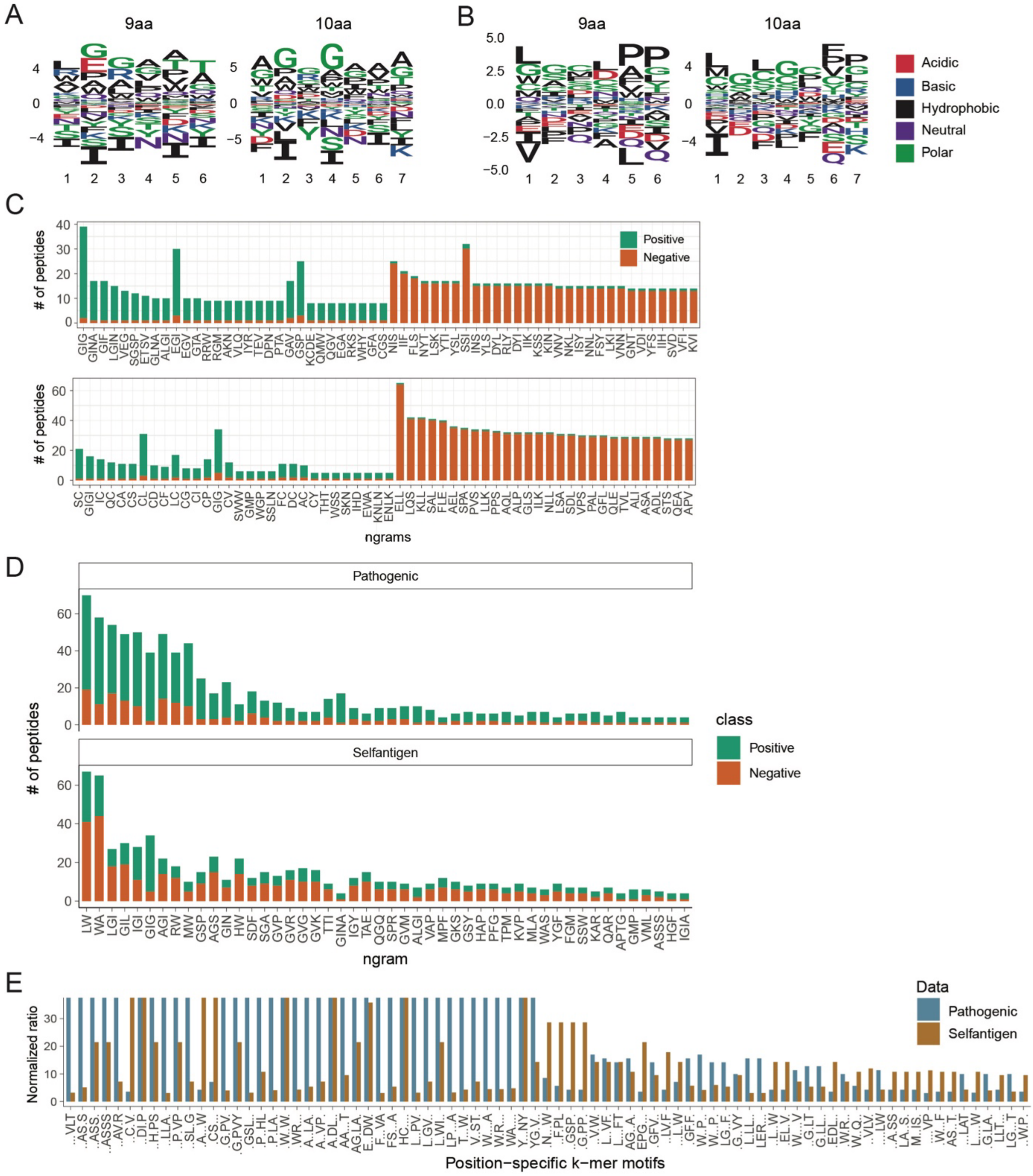

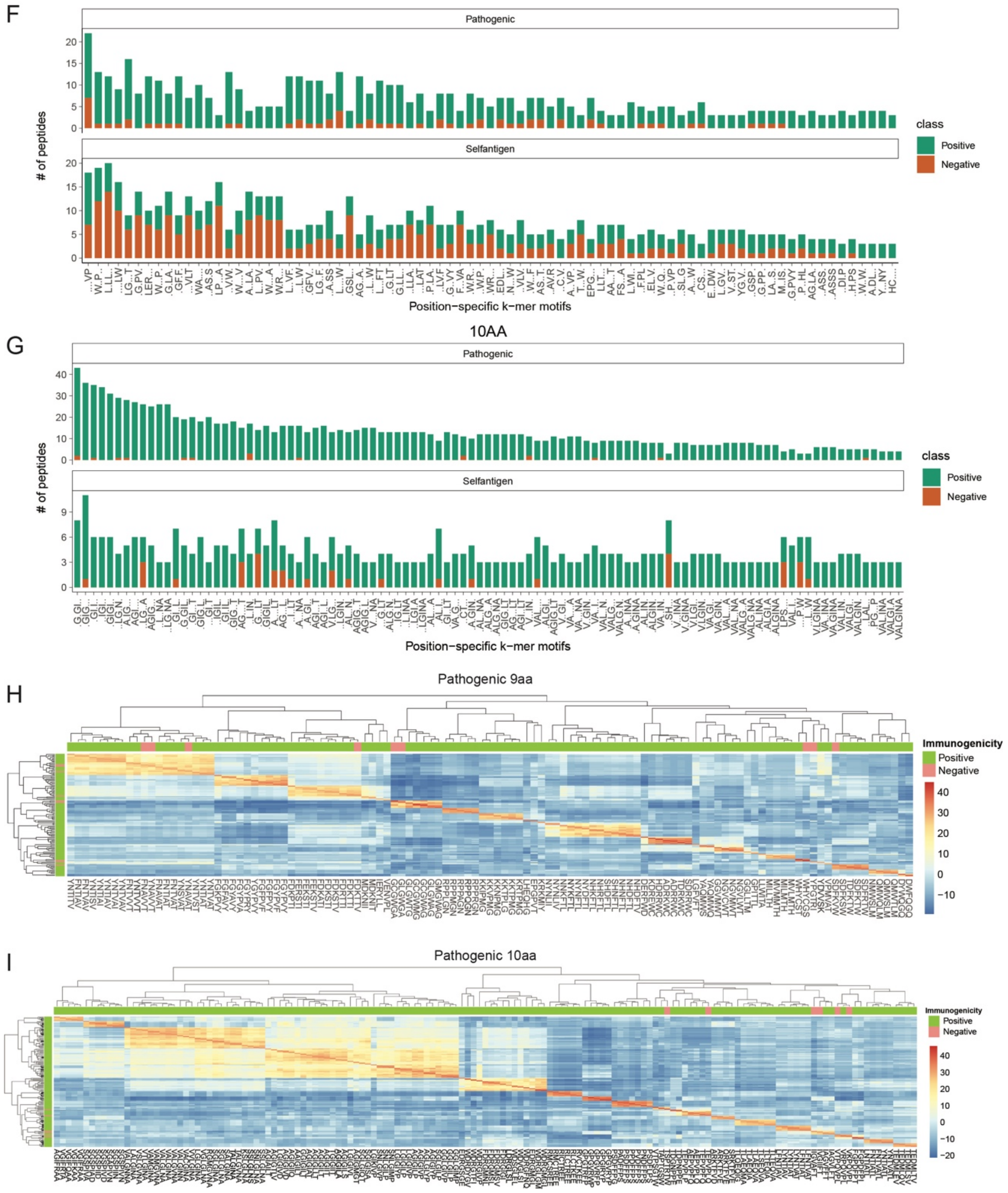

Supplementary Figure 6. Sequence patterns discriminating epitopes versus non-epitopes. A-B. Sequence logo of differential amino acid usage in contact positions of 9aa and 10aa peptides in pathogenic (A) and self-peptides (B). C. Top n-grams enriched (green) or depleted (orange) in epitopes for pathogenic (top) or self-antigens (bottom). D. Example of top n-grams found in both pathogenic and self-epitopes. Shown is the number of peptides containing the n-grams. E. Normalized ratio of shared position-specific k-mer motifs found from 9aa peptides enriched in both pathogenic and self-epitopes. F-G. Example of top epitope-enriched position-specific k-mer motifs found in both pathogenic and self-peptides for 9aa (F) and 10aa (G). Shown is the number of peptides containing the position-specific k-mer motifs. H-I. Clusters of peptides with high sequence similarity in contact positions, demonstrated by pairwise global alignment scores. Heatmap showing 9aa (H) and 10aa (I) pathogenic peptides having global alignment score ≥ 27 with ≥ 3 other peptides.

Fig S7

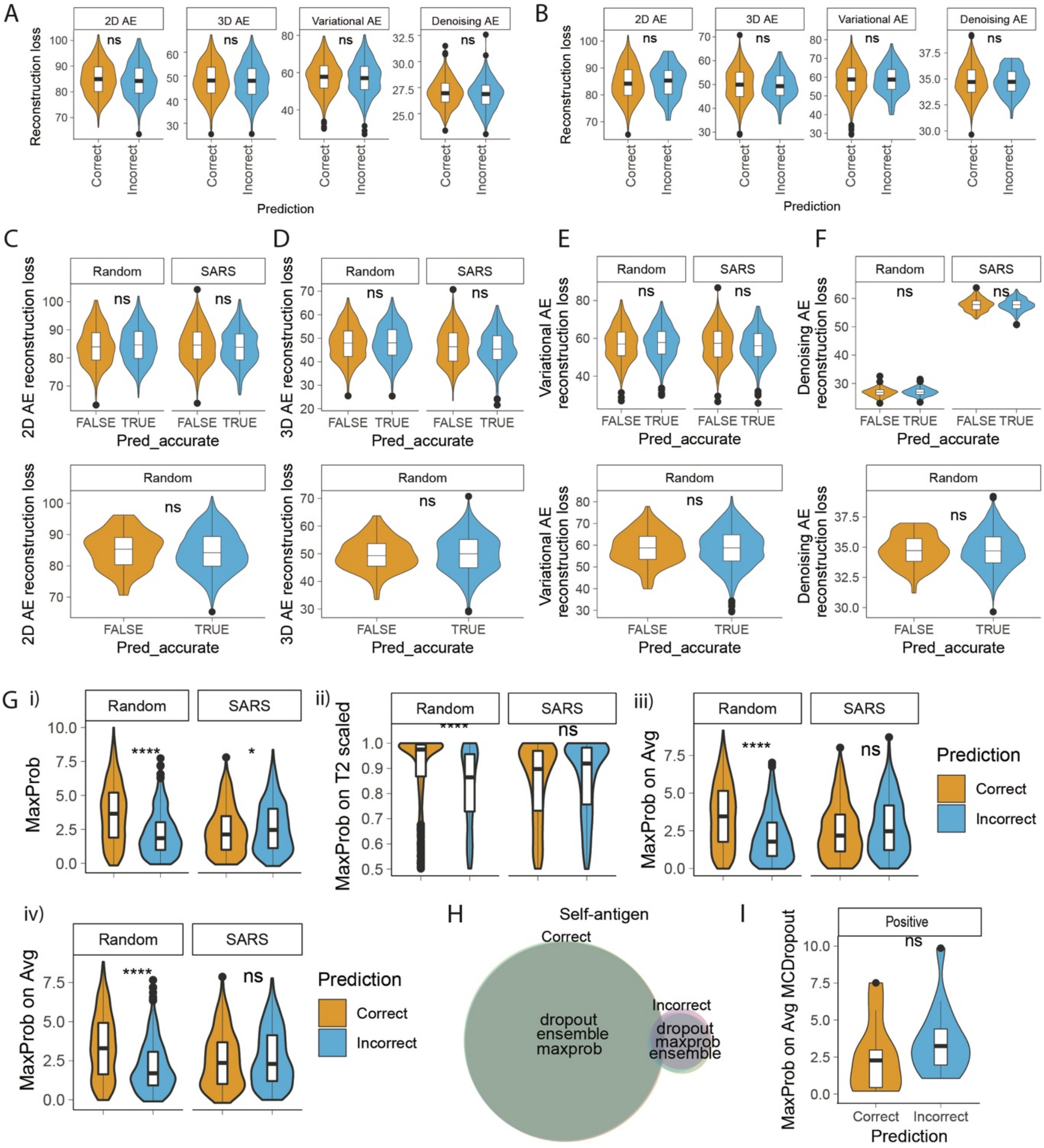

Supplementary Figure 7. OOD detection. A-B. Anomaly detection using autoencoders to discriminate correctly or incorrectly predicted pathogenic (A) and self- (B) peptides. Autoencoders included are 2-dimensional autoencoder (2D AE), 3-dimensional autoencoder (3D AE), variational autoencoder and denoising autoencoder. Statistical significance by p-values from Student's t-test. Ns: non-significant. We observed no significant difference in reconstruction loss between correctly and incorrectly predicted peptides, implying that the autoencoder-based methods cannot effectively identify OOD inputs. We speculated that unsupervised anomaly detection methods, such as reconstruction loss from autoencoders, were ineffective because 'species' was not the primary driver of peptide clustering at contact positions, which was often driven by the cognate TCRs to which they were bound. Likewise, density-based or distance-based methods that use sequence-representation similarity would not be able to detect OODs. C-F. Anomaly detection using autoencoders – 2D-AE (C), 3D AE (D),

variational autoencoder I and denoising autoencoders (F) for discriminating correctly vs. incorrectly predicted pathogenic (top) and self- (bottom) peptides. For SARS-2 peptides, non-SARS-2 peptides were used to train autoencoders to predict correctly vs. incorrectly predicted SARS-2 peptides. Statistical significance by p-values from Student's t-test. Ns: non-significant. G. Distribution of maximum softmax probability (MaxProb) (i), temperature scaling using different levels of temperatures that scale the logit values (ii), the maximum on average softmax probability over 10 ensembled models (MaxProb on Avg) (iii) and maximum on average softmax probability from Monte Carlo dropout iterations (iv) for randomly divided data (resemblance of 10-fold cross-validation) and cross-species data (i.e. model trained using non-SARS-CoV-2 peptides to predict SARS-CoV-2 peptides). The Monte Carlo models were reiterated 100 times with stochastic dropouts of 0.6. T: temperature. Statistical significance by p-values from Student's t-test. Ns: non-significant. H. Venn diagram comparing the self-antigen peptide predictions using different calibration metrics. I. Distribution of MCDropout values for glioblastoma cancer peptides predicted to be immunogenic using the TRAP self-antigen model.

Fig S8

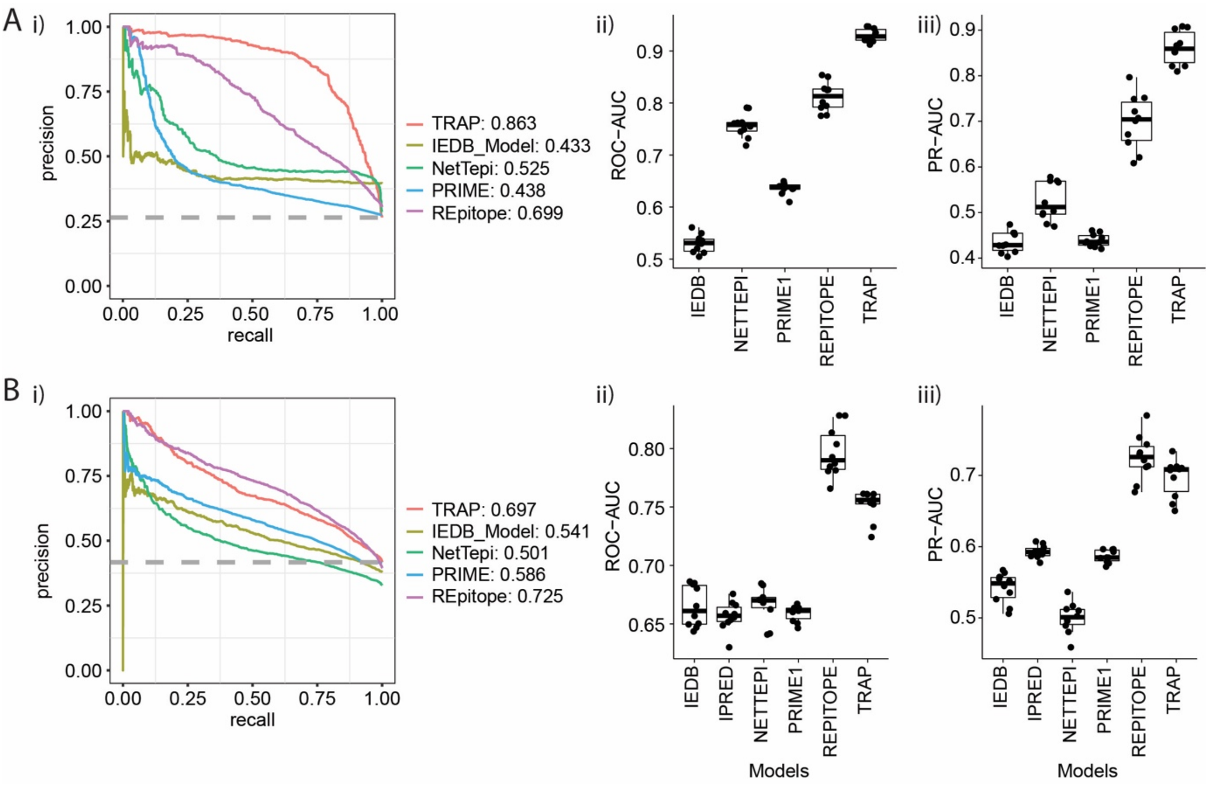

Supplementary Figure 8. Benchmark TRAP performance. A-B. Precision-recall (PR)-AUC and ROC-AUC values comparing the performance of TRAP with existing models, such as IEDB, iPred, NetTepi, PRIME, Repitope and DeepImmuno for self- (A) and pathogenic peptides (B).For ii-iii, each point represents ROC-AUC (ii) or PR-AUC (iii) value from one round of 10-fold cross-validations. Supplementary to Figure 6.
