# Supplementary material for: A robust deep learning workflow to predict CD8 + T-cell epitopes
Source: Genome Med. 2023 Sep 13;15:70. doi: 10.1186/s13073-023-01225-z (PMC10498576; doi:10.1186/s13073-023-01225-z)
Supplement: Supplementary file 2 — Additional file 2. Includes step-by-step tutorial to TRAP web application. [file 13073_2023_1225_MOESM2_ESM.pdf]

Tutorial for using TRAP web application

1. Choose whether to use pathogenic vs. self-antigen model

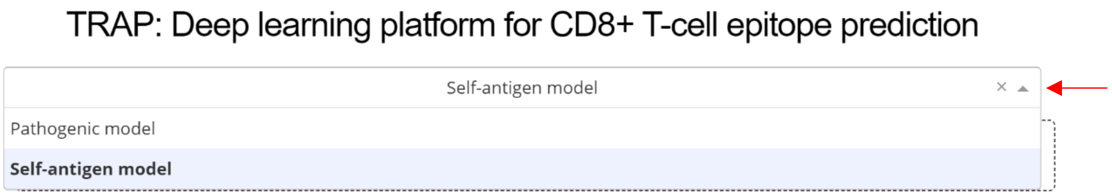

The pathogenic model predicts the immunogenicity of peptides derived from bacteria, viruses, or other pathogens. In contrast, the self-antigen model is intended to predict the immunogenicity of self-peptides in the context of cancer, autoimmunity, or other peptides derived from humans.

2. Insert test dataset

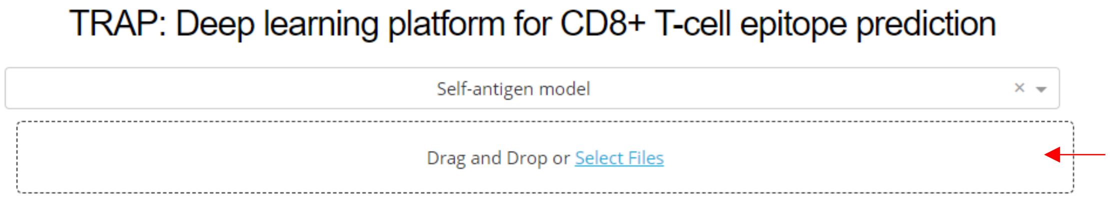

The minimal requirements of the test dataset include: 1) peptide sequences, and 2) their -log transformed NetMHCpan4.1 rank score for HLA alleles of interest. Please use the same column names as shown in the example dataset below.

Example input dataset:

Peptide	nlog2Rank
AAIESFVSV	1.493296513
ALAGGLYEY	-0.722728309
ALFCHQYDI	0.199089749
ALGALLILQL	-0.725348533
ALKSDFKLV	-0.480885503
ALQEEQNIL	0.982505621
ALQQLTTHM	0.050203631

3. Easy! The TRAP application will predict and plot results using pre-trained models!

TRAP: Deep learning platform for CD8+ T-cell epitope prediction

Self-antigen model ×

Drag and Drop or Select Files

Making predictions...

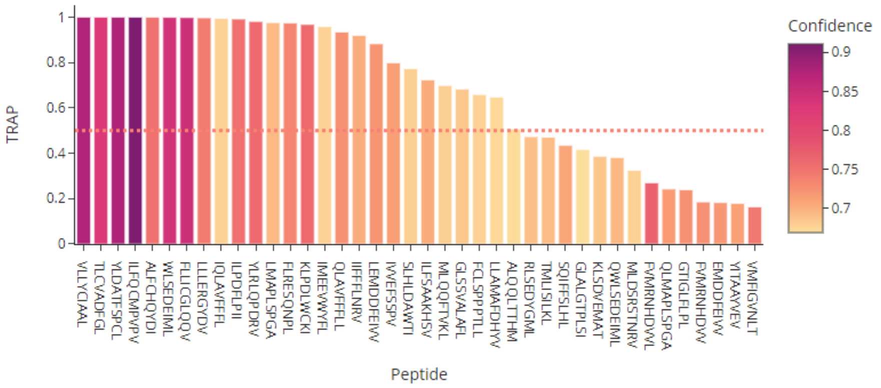

gbm_example_test_data.csv

EXPORT

Peptide	nlog2Rank	TRAP	MCDropout	Confidence
VLLYCIAAL	-0.614	0.99988	6.58	0.871
TLCVADFGL	-0.404	0.99987	5.362	0.834
YLDATFSPCL	-0.019	0.99985	6.662	0.874
ILFQCMPVPV	-0.568	0.99981	7.448	0.897
ALFCHQYDI	0.199	0.99947	2.636	0.753
WLSEDEIML	4.658	0.99886	5.756	0.846
FLLICGLQQV	0.907	0.99786	5.844	0.849
